# The Prevalence of Gastric Intestinal Metaplasia and Distribution of *Helicobacter pylori* Infection, Atrophy, Dysplasia, and Cancer in Its Subtypes

**DOI:** 10.1155/2015/434039

**Published:** 2015-11-09

**Authors:** Sehmus Olmez, Mehmet Aslan, Remzi Erten, Suleyman Sayar, Irfan Bayram

**Affiliations:** ^1^Medical Faculty, Department of Gastroenterology, Yuzuncu Yil University, 65080 Van, Turkey; ^2^Medical Faculty, Department of Internal Medicine, Yuzuncu Yil University, 65080 Van, Turkey; ^3^Medical Faculty, Department of Pathology, Yuzuncu Yil University, 65080 Van, Turkey; ^4^Dr. Ersin Arslan Public Hospital, Department of Gastroenterology, Gaziantep, Turkey

## Abstract

*Objectives*. Gastric intestinal metaplasia (IM) is frequently encountered and is considered a precursor of gastric adenocarcinoma. In the Van region of Turkey, gastric adenocarcinoma incidence is high but the prevalence of gastric IM is not known. *Helicobacter pylori* (*H. pylori*) infection is a main factor leading to atrophy, IM, and cancer development in the stomach. The aim of the current study was to investigate the prevalence of IM and its subtypes and the prevalence of *H. pylori* infection, atrophy, dysplasia, and cancer in gastric IM subtypes. *Materials and Methods*. This retrospective study was conducted on 560 IM among the 4050 consecutive patients who were undergoing esophagogastroduodenoscopy (EGD) with biopsy between June 2010 and October 2014. Clinical records and endoscopic and histopathologic reports of patients with IM were analyzed. *Results*. The prevalence of gastric IM was 13.8%. The prevalence of incomplete IM was statistically significantly higher than complete IM. Type III IM was the most frequent subtype. *Conclusions*. Gastric IM is a common finding in patients undergoing EGD with biopsy in this region. High prevalence of incomplete type IM, especially type III, can be associated with the high prevalence of gastric cancer in our region.

## 1. Introduction

The incidence of gastric cancer has declined over the past decades but gastric cancer is still the fourth most common cancer and second leading cause of cancer related death worldwide [1, 2]. Gastric cancer has two types: intestinal and diffuse types. The pathogenesis of the intestinal type of gastric cancer is related to precursor lesions such as chronic atrophic gastritis, intestinal metaplasia (IM), and adenoma/dysplasia. However, the pathogenesis of the diffuse type of gastric cancer is not well defined [3].

*Helicobacter pylori* (*H. pylori*) infection is associated with severe gastritis, chronic atrophic gastritis, and IM, as well as gastric cancer [4–6]. Severe atrophy accompanying IM is related to a particularly high risk in* H. pylori* infected patients [7]. Some clinical studies reported that IM improves or did not progress after* H. pylori* eradication therapy [8–10]. As a result,* H. pylori* eradication is one of the most promising approaches in gastric cancer prevention [11].

Gastric IM is defined as replacement of gastric mucosa by epithelium of intestinal morphology and is generally considered to be a precursor of gastric cancer [12]. Risk factors of IM are* H. pylori* infection, high salt intake, smoking, alcohol consumption, and chronic bile reflux [13]. Although there are several classifications of IM types, currently they are generally subclassified as complete (type I) or incomplete (types II and III) [14, 15]. The incomplete type of IM, particularly type III, has a higher gastric cancer risk than the complete type [13, 15].

The incidence of gastric cancer varies in different parts of the world [1, 2]. Although the incidence of gastric cancer in Turkey is not high, its incidence in the Van region is very high and gastric cancer is an important health problem [16, 17]. Gastric IM is known as an important risk factor for gastric cancer. As far as we know, no data is available regarding its prevalence in the Van region of Turkey.

The aim of the current study was to investigate the prevalence of IM and its subtypes and the prevalence of* H. pylori* infection, atrophy, dysplasia, and cancer in gastric IM. We also compared the relationship between* H. pylori* infection and IM subtypes.

## 2. Materials and Methods

### 2.1. Subjects

This retrospective study was conducted with 560 consecutive IM patients among the 4050 patients who were undergoing esophagogastroduodenoscopy (EGD), between June 2010 and October 2014, at the Department of Gastroenterology of the Yuzuncu Yıl University Medical Hospital.

Data including age, gender, indication for EGD, and endoscopic findings and pathology reports (histopathologic features, presence of associated chronic gastritis,* H. pylori* atrophy, dysplasia, and cancer) were retrieved from electronic medical records.

Indications for EGD are as follows: gastric cancer alarming symptoms or cancer screening, abdominal pain, anemia, dyspeptic symptoms, reflux, and follow-up of Barrett's esophagus and other premalignant lesions. If more than one EGD was performed with the same patient, only the first examination was included in the study.

The exclusion criteria were patients undergoing gastric surgery and upper gastrointestinal bleeding and patients with lacking data.

Esophagogastroduodenoscopy was performed after local 10% lidocaine sedation with a Fujinon video endoscope (EG 530 WR, Tokyo, Japan). Mean biopsies number was 3.7 ± 1.6 [1–12], and if lesions were suspected to be cancerous, extra biopsies were taken from the suspected area.

Biopsy specimens were evaluated for the presence of atrophic gastritis and IM.* H. pylori* was determined using histology testing. Pathologists of the Yuzuncu Yıl University Medical Hospital examined all the specimens.

All the endoscopically biopsied materials are fixed with Holland solution, after routine tissue follow-up processes embedded into paraffin blocks and stained with hematoxylin and eosin. Specimens are stained with periodic acid-Schiff/Alcian Blue (PAS/AB) at pH 2.5 stain combination and High Iron Diamine Alcian Blue (HID-AB) at pH 2.5 stain. Subtypes of IM are determined according to Filipe et al. classification [18].

The specimens were fixed in formalin and assessed for* H. pylori* (by Giemsa staining), the degree of neutrophil infiltration, IM (by staining with hematoxylin and eosin), atrophy, dysplasia, and other lesions.

IM was identified by replacement of glandular epithelium with goblet cells. IM was classified into complete (type I) or incomplete types (types II and III).

Type I includes mature absorptive cells and goblet cells, while the incomplete type secretes sialomucins. In type I IM, in PAS/AB stain at pH 2.5, the goblet cells are positively stained; the cylindrical cells between them show no reaction in both combinations and have markedly brush borders.

Type II has few or absent absorptive cells, presence of columnar “intermediate” cells in various stages of differentiation secreting neutral and acid sialomucins and goblet cells secreting sialomucins or, occasionally, sulfomucins, or both. In type II IM, in PAS/AB stain at pH 2.5, goblet cells are positively stained; cylindrical cells contain PAS positively stained granules; in HID/AB pH 2.5, cylindrical cells show no reaction.

Type III is formed by columnar intermediate cells secreting predominantly sulfomucins and goblet cells secreting sialomucins, sulfomucins, or both. If more than one subtype of IM was present, the specimen was classified based on the predominant IM phenotype. In type III IM, in PAS/AB stain at pH 2.5, goblet cells and cylindrical cells are positively stained with AB and in HID AB stain at pH 2.5 combination is positively stained.

The study protocol was approved by the ethics committee of our university.

### 2.2. Statistical Analysis

Descriptive statistics for continuous variables (characteristics) were presented as mean, standard deviation, and minimum and maximum values while they were count and percent for categorical variables. Chi-square test was used to determine linear association between categorical variables. In addition, Fisher's exact test was performed to compare two proportions in groups. Statistical significance level was considered as 5% and SPSS (version 13.0) statistical program was used for all statistical computations.

## 3. Results

Among the 4050 consecutive patients who had gastric biopsies performed, 560 were found to have gastric IM. Patient mean age was 57 ± 15 years (range: 17–98 years). There were 227 (40.5%) females and 333 (59.5%) males. The most frequent indications for EGD included dyspepsia (41.2%), abdominal pain (25.4%), and reflux symptoms (13.4%). The most frequent gastric endoscopic findings were gastritis (nonerosive, erosive, atrophic, etc.) (75.3%), ulcer (6.1%), and cancer (3.9%). Demographic features, indication for endoscopy, and endoscopic findings of the study population are shown in Table 1. The majority of patients were 41–70 years of age.

**Figure 1:**
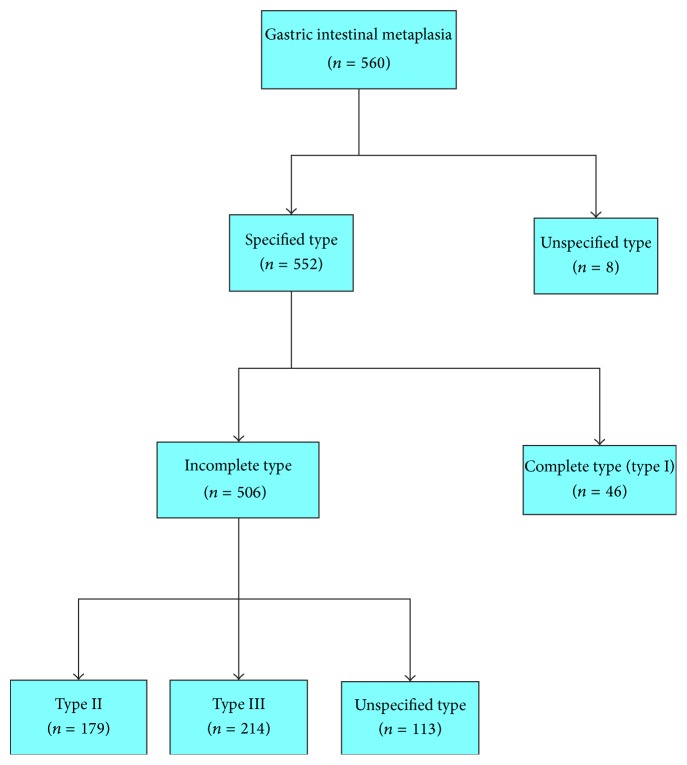
Flow diagram of intestinal metaplasia patients included in the study.

The prevalence of gastric IM was 13.8% at our institution. Eight of the 560 patients did not have an IM type (1.4%); 46 patients (8.2%) had complete type IM (Type 1). Of the 506 (90.4%) incomplete IM patients, 179 (32%) patients had type II, 214 (38%) patients had type III, and 113 (20.2%) patients had an unidentified subtype (Figure 1). The prevalence of incomplete IM was significantly higher than complete IM (*P* < 0.05).

Of the 46 complete IM patients, 6 had atrophy, 8 had dysplasia, and 1 had cancer. Of the 505 incomplete IM patients, 113 had atrophy, 72 had dysplasia, and 17 had cancer (Table 2).

Prevalence of* H. pylori* infection was 38.6% in gastric IM. The relationship between* Helicobacter pylori* status and types of IM was assessed. No significant difference in prevalence of complete type and incomplete type IM was found with* H. pylori* status (*P* > 0.05) (Table 3).

## 4. Discussion

In the model of gastric carcinogenesis,* H. pylori* plays a pivotal role in causing chronic active gastritis. Chronic* H. pylori* induces gastritis and may progress over years through the sequential stages of atrophic gastritis, IM, and dysplasia to gastric adenocarcinoma [19, 20]. An important risk factor for gastric cancer development is the presence of premalignant changes of the gastric mucosa, such as IM, atrophy, and dysplasia [4, 11, 21].

The prevalence of gastric IM and atrophy in the general population is known to vary around the globe, mostly depending on* H. pylori* status [7, 22]. IM is a well-known risk factor for the development of gastric cancer [21, 22]. Diagnosis of atrophic gastritis, IM, and dysplasia is often ignored in routine clinical practice [23]. There are no widely accepted guidelines for the management of gastric IM [24]. The American Society for Gastrointestinal Endoscopy (ASGE) and The European Society of Gastrointestinal Endoscopy and other European academic societies have developed evidence-based guidelines for the management of patients with gastric IM [11, 25]. ASGE guidelines for endoscopic surveillance for gastric IM cannot be uniformly recommended but surveillance may be beneficial for patients at increased risk of gastric cancer due to ethnic background or family history [25]. Recently, according to the published guidelines for endoscopic management of precancerous conditions and lesions in the stomach, endoscopic surveys should be offered to patients with extensive atrophy and/or IM without subtyping every three years [11]. Several studies demonstrated that endoscopic histological follow-up in patients with IM is able to detect gastric cancer at an early stage with a considerable mortality reduction and scheduled endoscopic control could be cost-effective in IM patients [26–28]. According to some authors, complete IM is associated with a lower risk of gastric cancer; therefore, in the absence of other risk factors for gastric cancer, patients with complete IM do not need long-term endoscopic surveillance [13]. Due to the high prevalence of gastric cancer and* H. pylori* infection in our region, we strongly recommend endoscopic surveillance of gastric IM without paying attention to subtype.

The prevalence of gastric IM was 13.8% in our study. The prevalence of gastric IM in the general population remains difficult to ascertain due to the asymptomatic nature of the lesion. There is a wide variation in the prevalence of gastric IM depending on the different methods used in studies and particularly the prevalence of* H. pylori* infection in the region. [22, 28]. Sonnenberg et al. conducted a large retrospective study of 78,985 patients undergoing EGD with biopsy across the United States and found that the prevalence of gastric IM was 7% [29]. Almouradi et al. reported that, among the 437 patients who had gastric biopsies performed, 66 were found to have gastric IM and they observed that the overall prevalence was 15% [30]. In Netherlands, the prevalence of IM in a large histopathology database was reported as 8% [21]. We think that the prevalence of IM may be higher in our region. This low prevalence of IM may derive from the insufficient number and inappropriate localization of biopsies.

In our study, the prevalence of incomplete type of IM (90.4%) was much higher than the complete type (8.2%). The type III IM was the highest subtype. Eriksson et al. reported that among 505 patients the total prevalence of IM was 19% and the prevalence of type III IM was 2.8%, type II IM was 4.4%, and type I IM was 11% [31]. Ozdil et al. reported that of 3301 consecutive dyspeptic patients 17.8% had IM. They observed that 86% had complete and 14% had incomplete IM [32].

The presence of incomplete type IM, especially type III, significantly increases the risk of gastric cancer as compared to type I and type II [28, 33–35]. High detection of incomplete type IM, particularly type III, may shed light on the high prevalence of gastric cancer in our region. The prevalence of incomplete type IM was considerably higher than in previous studies [36, 37].

Evidence on establishing the subtype of IM is limited and conflicting [24]. There are several problems when subtyping IM. There is the need for additional histochemical techniques and these techniques are far from being standardized. The current technique uses reagents such as High Iron Diamine Alcian Blue staining, which are toxic and potentially carcinogenic. Pathologically, subtyping is sometimes difficult because IM have a multifocal distribution within the stomach and IM areas may be small. As a result, sampling errors may be unavoidable. Also, different subtypes of IM frequently coexist. When there are different subtypes, it is unclear which should be considered for categorizing [12, 34]. Perhaps due to these reasons, in our study, a distinction of complete and incomplete types in eight of 560 patients and a distinction of type II and type III in 113 patients could not be taken into account.

In our study, we observed that gastric IM was higher in older than in younger patients. Several studies revealed that age ≥50 years was an independent risk factor for IM, which is consistent with previous studies reporting that the incidence of IM increases with age [25, 29, 36].

In a study regarding* H. pylori* infection in Turkey, Özden et al. found* H. pylori* positivity to be 81% in the general population [38]. In our study, the prevalence of* H. pylori* infection was 38.6% in gastric IM and no significant difference in prevalence of complete type and incomplete type IM was found with* H. pylori* presence. With and without atrophy, IM may cause lower diagnostic accuracy of* H. pylori* with histologic examination [13]. In addition, decreased sensitivity of the rapid urease test is observed in subjects taking proton pump inhibitors, so other diagnostic tests, including serology or stool antigen, should be considered [13, 37].* H. pylori* eradication is one of the most promising approaches in gastric cancer prevention [11]. However, whether gastric atrophy and IM are reversible after the eradication of* H. pylori* remains controversial [22, 39], since in some studies eradication of* H. pylori* has not been effective in IM regressions [8–10, 26]. However, several studies have shown that eradication of* H. pylori* infection significantly retarded progression of gastric IM and therefore* H. pylori* eradication has been proposed [40–43].

This retrospective study design has several potential limitations. Firstly, biopsies may not have been taken in adequate number and from the same part of the stomach. Secondly, results of the study may be affected by the lack of medical history such as information about proton pump inhibitors or antibiotic usage in some patients. Finally, diagnosis of* H. pylori* infection was only made by histological examination.

In conclusion, this study indicated that gastric IM is a common finding in patients undergoing EGD with biopsy. In addition, we found that the prevalence of incomplete IM was statistically significantly higher than complete IM. In our study population, type III IM was the most frequent subtype. High prevalence of the incomplete type and notably type III may be associated with a high prevalence of gastric cancer in our region.* H. pylori* was detected in 38.6% of gastric IM patients in Turkey. To further establish the relationship between IM and gastric cancer in our region, large prospective, randomized, and multicenter studies are needed.

## Figures and Tables

**Table 1:** Demographic features, indication for EGD, and EGD findings of patients with intestinal metaplasia.

	(*n* = 560)
Sex	
Male	227 (40.5%)
Female	333 (59.5%)
Age (years)	57.0 ± 15.3 (17–98)
Age groups	
<31	29 (5.2%)
31–40	56 (10%)
41–50	115 (18.8%)
51–60	144 (25.7%)
61–70	113 (20.2%)
71–80	87 (15.5%)
>80	26 (4.6%)
Indication for EGD	
Dyspepsia	231 (41.2%).
Abdominal pain	145 (25.9%)
GERD	75 (13.4%)
Cancer screening	29 (5.2%)
Others	80 (14.3%)
EGD findings	
Gastritis (nonerosive, erosive, atrophic, etc)	378 (67.5%)
Gastric ulcer	34 (6.1%)
Gastric cancer	22 (3.9%)
Others	126 (22.5%)

GERD: gastroesophageal reflux disease; EGD: esophagogastroduodenoscopy.

**Table 2:** Prevalence of atrophy, dysplasia, and cancer in types of intestinal metaplasia.

	Complete IM (*N* = 46) *n* (%)	Incomplete IM (*N* = 506) *n* (%)	Fisher's exact test *P* value
Atrophy	6 (13%)	113 (22.3%)	0.080
Dysplasia	8 (17.4%)	72 (14.2%)	0.586
Cancer	1 (2.2 %)	17 (3.4%)	0.999

IM: intestinal metaplasia; *P* < 0.05 statistically significant.

**Table 3:** Relationship between *Helicobacter pylori* status and types of intestinal metaplasia.

*Helicobacter Pylori *	Complete IM (*N* = 46) *n* (%)	Incomplete IM (*N* = 506) *n* (%)	Fisher's exact test *P* value
Negative	25 (54.3%)	314 (62.1%)	0.314
Positive	21 (45.7%)	192 (37.9%)	

IM: intestinal metaplasia; *P* < 0.05 statistically significant.
